# Genome-Wide Identification of Long Non-Coding RNAs and Their Potential Functions in Poplar Growth and Phenylalanine Biosynthesis

**DOI:** 10.3389/fgene.2021.762678

**Published:** 2021-11-15

**Authors:** Lei Zhang, Xiaolan Ge, Jiujun Du, Xingqi Cheng, Xiaopeng Peng, Jianjun Hu

**Affiliations:** ^1^ State Key Laboratory of Tree Genetics and Breeding, Key Laboratory of Tree Breeding and Cultivation of National Forestry and Grassland Administration, Research Institute of Forestry, Chinese Academy of Forestry, Beijing, China; ^2^ Collaborative Innovation Center of Sustainable Forestry in Southern China, Nanjing Forestry University, Nanjing, China

**Keywords:** lncRNA–mRNA, poplar, phenylalanine biosynthesis, xylem, hormone

## Abstract

Poplar is an important bioenergy tree species. lncRNAs play important roles in various biological regulatory processes, and their expression pattern is more tissue-specific than mRNAs. In this study, P. deltoides “Danhong” (Pd) and P. simonii “Tongliao1” (Ps) with different growth rates and wood quality were used as experimental materials, and the transcriptomes of their shoot apical meristem, xylem, and phloem were sequenced. Furthermore, high-throughput RNA sequencing analysis revealed that the expression patterns of genes and lncRNAs are different between the two genotypes. 6,355 lncRNAs were identified. Based on target prediction, lncRNAs and target genes were involved in ADP binding, oxidoreductase activity, phenylpropanoid biosynthesis, and cyanoamino acid metabolism. The DElncRNAs in two poplars were co-expressed with transcription factors and structural genes of lignin and flavonoid pathways. In addition, we found the potential target lncRNAs of miRNA. This result provides basic evidence for a better understanding of the regulatory role of lncRNAs in regulating phenylalanine molecular pathways and wood formation.

## Introduction

Plants are unique in their ability to continuously produce new organs throughout their life cycles. The process of continuous organogenesis depends on the activity of pluripotent cells (Xue et al., 2020). In trees, this mainly refers to the shoot apical meristem (SAM) affecting high growth and the vascular cambium affecting radial growth (Elo et al., 2009). The SAM generates leaves, stems, and floral organs throughout the lifespan of higher plants (Ha et al., 2010; Xue et al., 2020). The cambium differentiates into xylem and phloem, determined cell types, and cell layers in the secondary xylem (Ye and Zhong, 2015). These complex processes are easily regulated by plant hormones, transcription factors (TFs), miRNAs, and lncRNAs (J. Zhang G. et al., 2018; Xue et al., 2020).

lncRNAs are non-coding transcripts longer than 200 nucleotides (nts), including intergenic, intronic, sense, and antisense types (Ma et al., 2013). Compared with protein-coding genes (PCgenes), most lncRNAs are less conserved between species, lower expression levels, and stronger tissue-specific expression patterns (Liu et al., 2012; Zhou et al., 2017; Xu et al., 2018). lncRNAs can regulate genes expression at transcriptional, posttranscriptional, and epigenetic levels and play an important role in genomic imprinting, chromatin remodeling, transcriptional activation, transcriptional interference, and cell cycle (Wang and Chekanova, 2017; Sun et al., 2018). With the continuous development of resequencing technology, lncRNAs of more and more species have been identified. They are widely involved in embryo development, seed formation, flower development, secondary growth of wood, and abiotic stress response (Zhou et al., 2017; Severing et al., 2018; Xu et al., 2018; Jiang et al., 2019; Wu et al., 2019). For example, lncRNAs play a potential regulatory role in endosperm and embryo development of castor bean (Xu et al., 2018). *COOLAIR* and *COLDAIR* play an important role in regulating vernalization in *Arabidopsis* (Heo and Sung, 2011). *FLINC* lncRNA participates in ambient temperature–mediated flowering time of *Arabidopsis* (Severing et al., 2018). lncRNAs influence the formation of tension wood by regulating *ARF*s in *Catalpa bungei* (Xiao et al., 2020). lncRNAs are widely involved in the secondary growth, GA response, heat tolerance, low nitrogen stress, salt stress, and other life processes of poplar (Chen et al., 2016; Tian et al., 2016; Ci et al., 2019; Ma et al., 2019; Song et al., 2020).

*Populus* is often used as a short rotation coppice (SRC) and bioenergy tree species all over the world because of its fast growth and reduced inhibitory extract from wood fermentation during bioenergy conversion (Guerra et al., 2013; Zhang et al., 2020b). So, the growth rate determines the economic benefit and the output of biomass energy. There are significant differences in the growth rate of *P*. *deltoides* “Danhong” and *P*. *simonii* “Tongliao1.” *P*. *deltoides* “Danhong” is a southern poplar characterized by fast growth and insect resistance (Zhang et al., 2008). *P*. *simonii* is a native tree species in northern China; although the growth rate is slow, it is resistant to cold and drought (Wei et al., 2013).

In order to identify the regulation mechanism of growth and wood property differences and provide theoretical basis for breeding new germplasm with fast growing ability, we selected *P*. *deltoides* “Danhong” and *P*. *simonii* “Tongliao1” as experimental materials and identified the important lncRNAs that may be involved in growth regulation by sequencing. In this study, the sequencing of lncRNA libraries was constructed from the SAM, phloem, and developing xylem of *P*. *deltoides* “Danhong” and *P*. *simonii* “Tongliao1.” *P*. *tricorcarpa* was used as the reference genome for the identification of lncRNA. We identified a total of 6,355 lncRNAs, of which 2,454 were sense_overlapping lncRNAs, 2,004 were lincRNAs, and 1,897 were antisense lncRNAs. The functional prediction of lncRNAs and their expressions as involved in wood development were examined. We investigated putative functional lncRNA candidates by differential expression analysis and co-expression network construction during SAM and xylem development. The important miRNA–lncRNA pairs in phenylalanine biosynthesis and hormone transduction were identified.

## Materials and Methods

### Plant Materials

One-year-old *P*. *deltoides* “Danhong” (Pd) and *P*. *simonii* “Tongliao1” (Ps) were cultivated in the experimental field of the Chinese Academy of Forestry, Beijing, China (116.256°E, 40.007°N). We collected shoot apical meristem (SAM, Pd_S and Ps_S) and scraped phloem (inside of the bark, Pd_P and Ps_P) and developing xylem (newly formed xylem cells about 2–3 mm, Pd_X and Ps_X) from Pd and Ps, respectively, at diameter breast height (DBH) during the fast-growing period (July 20, 2019). Each tissue had three biological replicates. The samples (2 genotypes × 3 tissues × 3 biological replicates) used for RNA extraction were frozen immediately in liquid nitrogen and stored at −80°C. Shoot tips and cuneiform blocks (phloem, cambium, and xylem) at DBH for histologic analysis were fixed in a mixture of formalin, glacial acetic acid, and 70% ethanol in the ratio 5:5:90 vol.; FAA under vacuum for at least 24 h.

### Histologic Analysis

Stem pieces were embedded with Spurr resin as described by Zhang et al. (2020d). A cross section of 4 µm thick was obtained from the stem by Leica M205FA, while the SAM sections of 40 μm were obtained using a rotary microtome (Leica VT1200S, Wetzlar, Germany). The sections were stained using 0.05% toluidine blue O (TBO) and were examined with a microscope (Zeiss). The number and diameter of vessel cells in the same area (1,260 × 980 µm) were counted by ImageJ (version 1.8.0).

### Wood Property Determination

In order to understand the difference of wood properties between Pd and Ps, we measured the plant height and ground diameter and collected the stems to measure the wood properties including basic density, fiber length, fiber width, microfibril angle, cellulose, holocellulose, and lignin content of 1-year-old trees in December 2019. The basic density was determined by using the drainage method. A 10-cm-high wood segment was cut from the base of the trunk without bark and pith. It was softened by heating in 30% nitric acid and a small amount of potassium chlorate and converted into wood pulp by forced oscillation. The length and width of the fiber were measured 50 times using Shyygx Measure 2.0. Wood flour (40–60 mesh) from a 5-cm basal stem segment was used to determine the chemical composition. The content of holocellulose and lignin was calculated according to Chinese standards GB/T 2677.10-995 and GB/T 2677.8-1994, respectively. To evaluate the content of cellulose, the specimens were extracted with a mixed solvent of nitric acid and ethanol (v/v = 1/1) (Zhan et al., 2015). Three replicates were performed for each variety.

### Total RNA Isolation, Library Construction, and Illumina Transcriptome Sequencing

Total RNA was isolated from the 18 samples (SAM, phloem, and developing xylem) using the RNAprep Pure Plant Plus Kit (TIANGEN, China). An index of the reference genome (*P. trichocarpa* v3.0) was built using HISAT2 (Kim et al., 2015). StringTie was used to calculate FPKMs of both lncRNAs and coding genes in each sample (Pertea et al., 2015). Sequencing data are available in NCBI SRA database (SRA number: SRP2343030 to SRR13961247).

### lncRNA Identification

We used four filtration steps to identify lncRNAs from the transcriptome assembly: 1) Transcripts with an exon number ≥2 and length ≥200 bp were selected. 2) CuffCompare software was used to screen out transcripts that overlap with the database annotation exon field. 3) Evaluation of Coding Potential Calculator (CPC) (Kong et al., 2007), Coding Potential Assessment Tool (CPAT) (Wang et al., 2013), and Coding-Non-Coding Index (CNCI) (Sun et al., 2013) was carried out to screen whether there is coding potential. 4) They were referred to the the Hugo Gene Nomenclature Committee (HGNC) to name the novel_lncRNA of this analysis (Wright, 2014).

### Prediction of the Target Gene

Two methods were used to predict lncRNA target genes. *Cis* target genes were predicted according to the location relationship between lncRNA and mRNA, and the screening range was within 10 kb (Jia et al., 2010). Co-expression–related target genes were predicted according to the expression correlation between lncRNA and mRNA, and the screening condition is that the correlation coefficient is greater than 0.95 and *p*-value< 1.68E-09. The mRNA–lncRNA regulatory network was further modeled and visualized using Cytoscape 3.8 (Otasek et al., 2019).

In order to identify lncRNAs that may be used as precursors of miRNAs, we compared the published miRNAs of *P. trichocarpa* in miRBase (http://www.mirbase.org/search.shtml) with lncRNAs. lncRNAs as targets of miRNAs were predicted by Novomagic, a free online platform for data analysis (https://magic.novogene.com).

### Differential Expression Genes and Functional Analysis

To identify the differential expression of lncRNAs and mRNAs between Pd and Ps, we performed the read count of pair-wise comparisons (Pd_S vs Ps_S, Pd_P vs Ps_P, Pd_X vs Ps_X, Pd_S vs Pd_P, Pd_S vs Pd_X, Pd_P vs Pd_X, Ps_S vs Ps_P, Ps_S vs Ps_X, Ps_P vs Ps_X) by DESeq R package with a q-value < 0.05 (Love et al., 2014). Finally, those putative *cis*- and coexpression-targets of lncRNAs were analysed using Gene Ontology (GO) analysis (Ashburner et al., 2000; Conesa et al., 2005), and KEGG (Kyoto Encyclopedia of Genes and Genomes) enrichment of DE genes was performed based on a corrected *p*-value < 0.05.

### Quantitative Real-Time (qRT)-PCR and Correlation Analysis of Expression Trends

We selected three DELs and three DEGs from the results of the transcriptional analysis and confirmed them through qRT-PCR. *PtrActin* and *PtrUBQ* were used as internal reference genes (Wang et al., 2020a) (Supplementary Table 1). Real-time PCR was conducted on a LightCycler 480 (Roche, Basel, Switzerland) using the SYBR Premix Ex Taq™ Kit (Takara, Dalian, China). The relative expression levels of the genes were calculated using the 2^−ΔΔCT^ method (Livak and Schmittgen, 2001), and the data are presented as mean ± SD from three independent biological replicates.

## Results

### Differences in Growth and Wood Properties Between Pd and Ps

**FIGURE 1:**
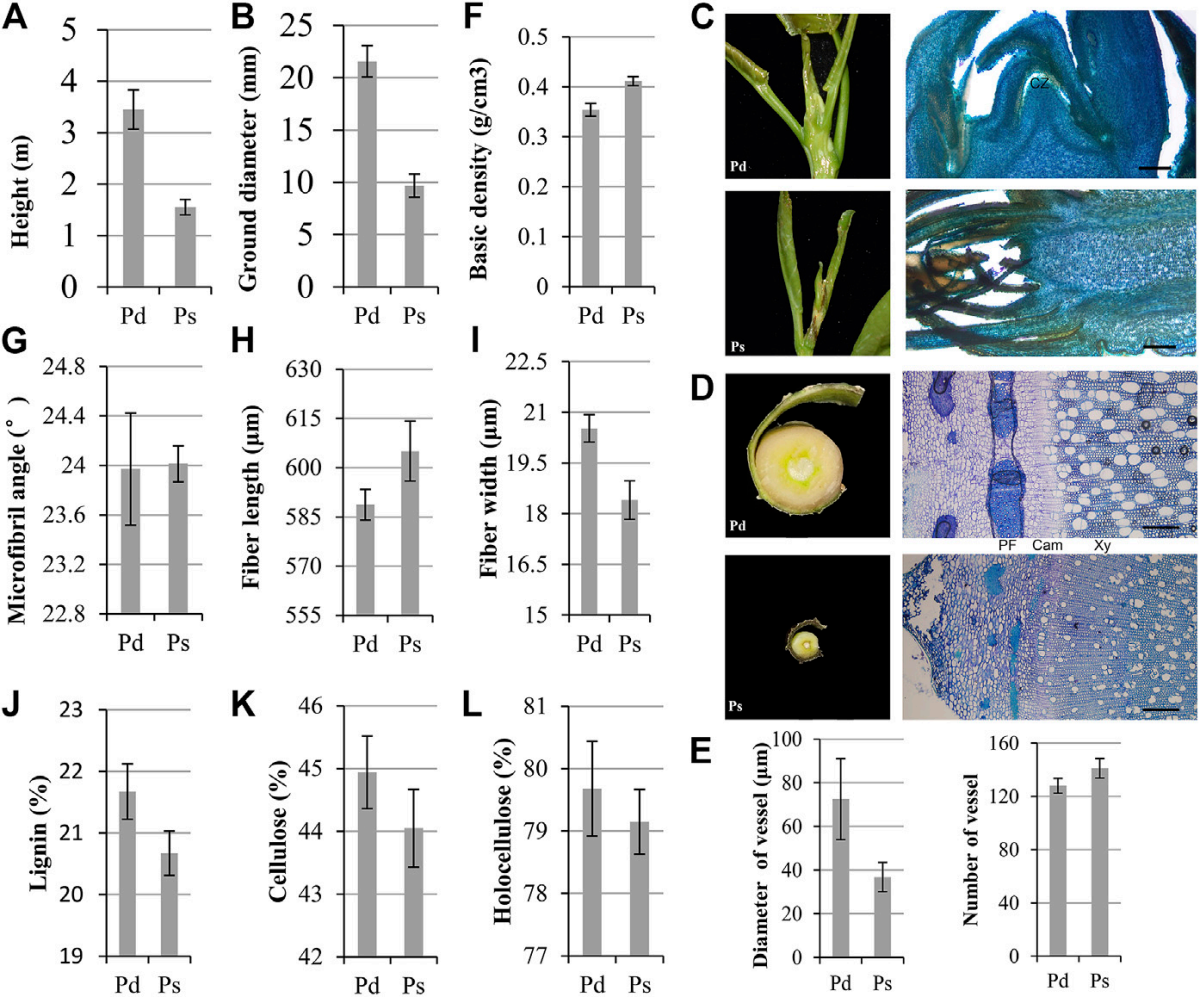
Phenotypic traits of *P*. *deltoides* “Danhong” (Pd) and *P*. *simonii* “Tongliao1” (Ps). Annual plant height **(A)** and ground diameter **(B)** of Pd and Ps. **(C)** Shoot apical meristem (SAM) image and section of Pd and Ps. Scale bars = 500 μm. **(D)** Cross sections of phloem–xylem region from 1-year-old trees. Scale bars = 200 μm. **(E)** Vessel size and number of vessel in the same area of Pd and Ps. CZ, central zone; PF, phloem fiber; Cam, cambium; Xy, xylem. Basic density **(F)**, microfibril angle **(G)**, fiber length **(H)**, fiber width **(I)**, lignin **(J)**, cellulose **(K)**, and holocellulose **(L)** content of Pd and Ps. Mean ± SD from three biological replicates.

Wood is the secondary xylem of trees, mainly composed of cellulose, hemicellulose, and lignin. All xylem cell types first undergo secondary cell wall (SCW) thickening and programmed cell death (Zhong and Ye, 2015; Zhang et al., 2020d). The plant height and ground diameter of annual Pd were significantly higher than those of Ps (Figures 1A,B). The SAM is an important regulatory site of plant height growth, and phloem and developing xylem are important parts of plant radial growth. The slice results showed that the SAM was surrounded by young leaves, and the SAM of Pd was conical and convex, while the Ps was flat, which suggested that the growth point of Ps might not be obvious enough, which caused the high growth to be slower (Figure 1C). The radial section of the stem showed that the phloem of Pd had wider phloem fibers, and the cambium was more obvious (Figure 1D). The average diameter of the vessel cells in the xylem of Pd was 72.5 μm, which was significantly larger than that of Ps (Figures 1D,E). We further determined the quality of wood. The basic density of basic Pd was less than Ps (Figure 1F), and there was no significant difference in the microfibril angle (Figure 1G). Compared with Ps, the fiber of Pd is short and thick (Figures 1H,I). Further material property determination found that the content of the three major elements of Pd is higher (Figures 1J–L).

### Identification of lncRNAs From SAM, Phloem, and Developing Xylem RNA-Seq Datasets

As an important fast-growing tree, it is very important to understand the molecular pathways of growth and development of poplar. After trimming adapters and removing low-quality and contaminated reads, in total, 246.24 Gb clean data were obtained from 18 libraries, with an average Q30 of 93.00% (Supplementary Table 2). Finally, we identified 6,355 lncRNAs, with protections of 2,454 sense_overlapping lncRNAs, 2,004 of lincRNAs, and 1,897 of antisense lncRNAs (Table S3).

**FIGURE 2:**
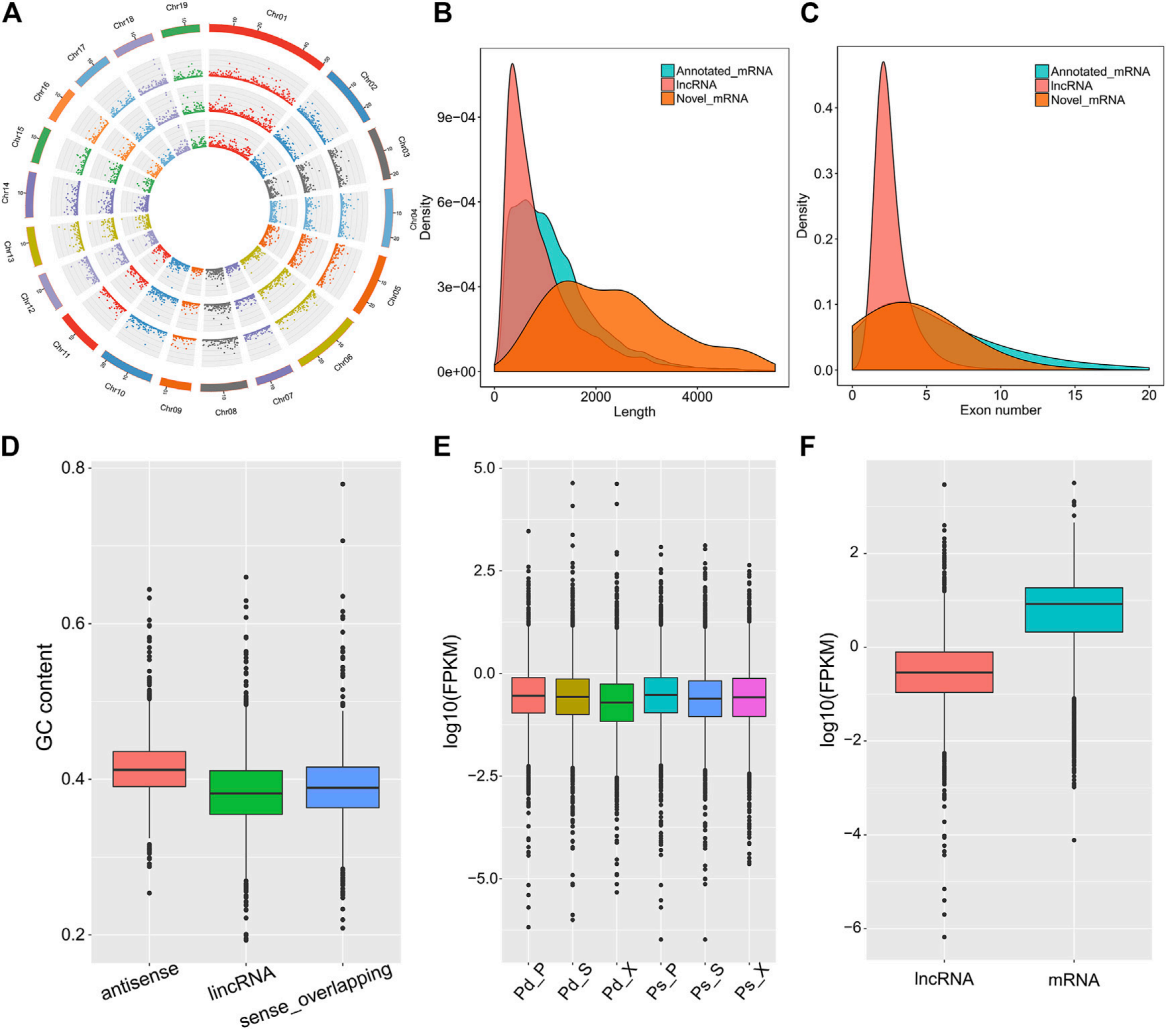
Characterization of cassava lncRNAs in Pd and Ps. **(A)** Distribution of lncRNAs along each chromosome for the SAM, phloem, and xylem in Pd from inside to outside. Distributions of length density **(B)** and exon numbers **(C)** in mRNAs, novel mRNAs, and novel lncRNAs. **(D)** GC content of lncRNAs. **(E)** Fragments per kilobase per million read (FPKM) values of lncRNAs in different samples. **(F)** Comparison of the expression levels of lncRNA and mRNA.

In order to analysis the characteristics of these lncRNAs, we evaluated the distribution of chromosome location, transcript length, exon number, and expression level of lncRNAs. In general, lincRNA, sense_overlapping, and antisense lncRNAs were evenly distributed on 19 chromosomes, although they had different emphases (Figure 2A). The average length of lncRNAs was 990 bp, and about 63.4% contained two exons (Figures 2B,C). Antisense lncRNAs ranged in length from 201 to 9,830 bp, and the average was 940 bp. lincRNAs ranged between 201 and 4,734 bp (average = 783 bp), and average length of sense_overlapping lncRNAs was 1,197 bp. The GC content of antisense lncRNA was 41.36%, which was significantly higher than that of lincRNAs and sense_overlapping lncRNAs (Figure 2D). For expression levels, the lncRNA expression levels were different and showed fewer average counts (FPKM = 4.44) than the coding transcripts (FPKM = 18.96) (Figures 2E,F).

**FIGURE 3:**
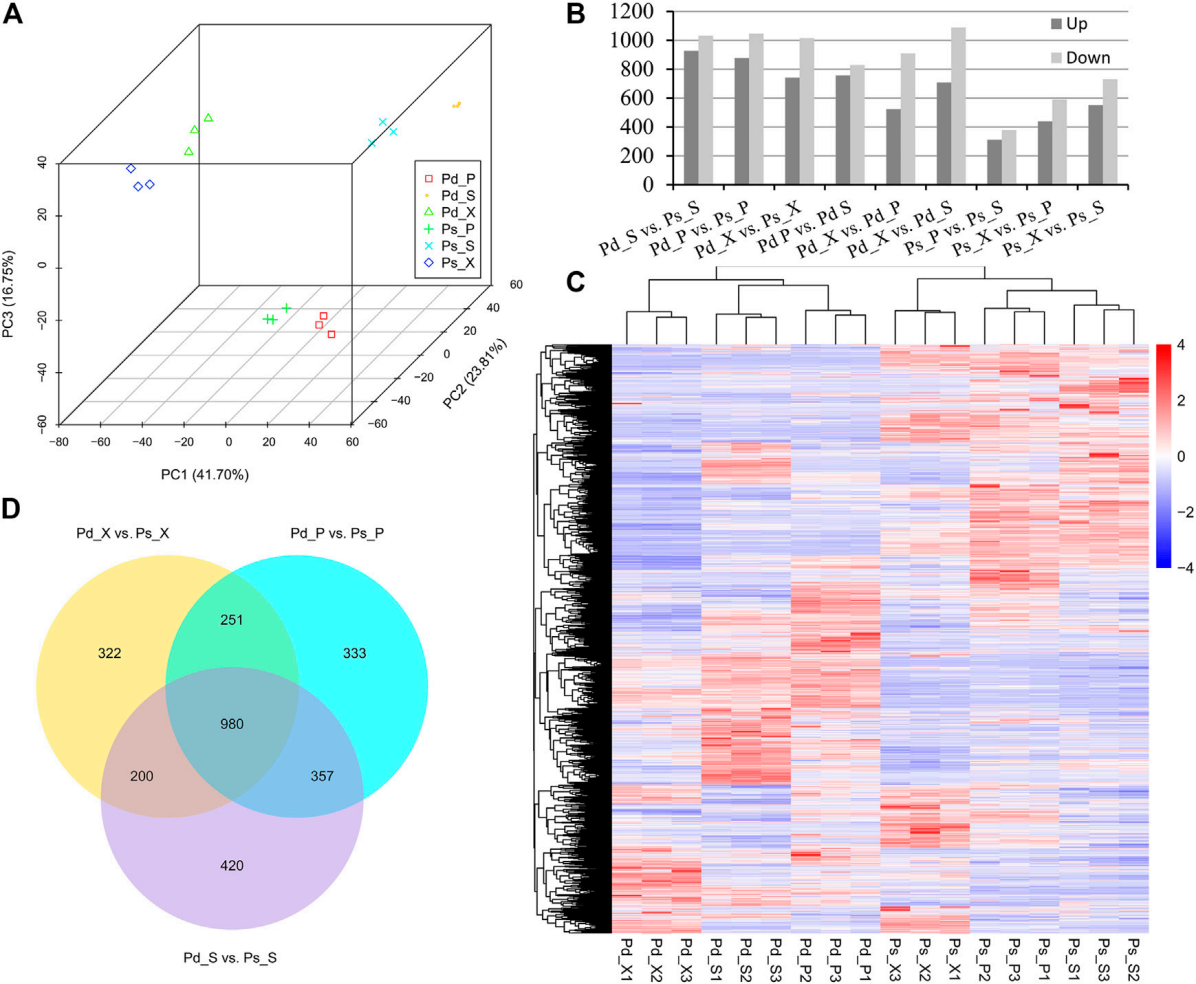
Differentially expressed (DE) lncRNAs identified in pair-wise comparison of 18 samples. **(A)** Principal component analysis (PCA) 3D of the expressed genes showing sample separation. **(B)** Number of DElncRNAs in different comparisons. **(C)** Heat map of DElncRNAs in the two genotypes. **(D)** Venn diagram showing DElncRNAs of Pd_X_vs_Ps_X, Pd_P_vs_Ps_P, and Pd_S_vs_Ps_S.

A principal component analysis (PCA) plot of the whole dataset revealed a sequential order of the different samples. The results showed that the SAM, xylem, and phloem of the two species were clustered into three groups, and the similarity of lncRNAs in tissues was greater than that between genotypes (Figure 3A).

### Differentially Expressed Anaylsis Between Pd and Ps

In order to further analyze whether these genes were differentially expressed between the two genotypes and different tissues, nine comparative combinations were carried out. Finally, 3,572 differentially expressed (DE) lncRNAs and 27,582 DEmRNAs were obtained. Among them, the DElncRNAs of Pd_S vs Ps_S were the highest in number, including 1957 lncRNAs (Figures 3B,C; Supplementary Figure 1). DEmRNAs participated in molecular functions such as “ADP binding” and “catalytic activity” (Supplementary Figure 1C). We also compared the DElncRNAs in three tissue difference genes between the two genotypes, and there were 980 DElncRNAs in the three comparison combinations (Pd_ X vs Ps_ X, Pd_ P vs Ps_ P and Pd_ S vs Ps_ S) and 322, 333, and 420 specifically expressed lncRNAs in the SAM, xylem, and phloem, respectively (Figure 3D).

### Enrichment Analysis of lncRNAs With a Potential Regulatory Function

Since lncRNAs play important roles in regulating gene expression, identification and analysis of their target genes may help us explore their potential functions. We calculated and predicted 13,932 co-localization pairs consisting of 3,413 lncRNAs and 10,627 RNAs and identified 72,038 co-expression pairs consisting of 1,975 lncRNAs and 11,709 RNAs (Tables S4, S5). To further analyze the function of these lncRNAs, we performed GO and KEGG analyses on their target genes. The colocation target genes of DElncRNAs were mainly enriched in 60 GO terms such as “ADP binding” and “nucleoside binding” (Supplementary Figure 2). Some target genes were enriched in the photosynthesis pathway, including 39 lncRNAs and 68 mRNAs. For example, TCONS_00135489 showed the same trend as its target, and the expression level of related genes was high in the SAM (Supplementary Figure 3). And 3,489 genes were found by co-location and co-expression of lncRNAs. They participated in immune response, cell death, purine nucleotide binding, and ATP binding progress, and the KEGG enrichment analysis shows that they were enriched in cytochrome P450, chalcone synthase, and phenylalanine ammonia-lyase which were important parts of phenylpropanoid biosynthesis (Figure S4).

**FIGURE 4:**
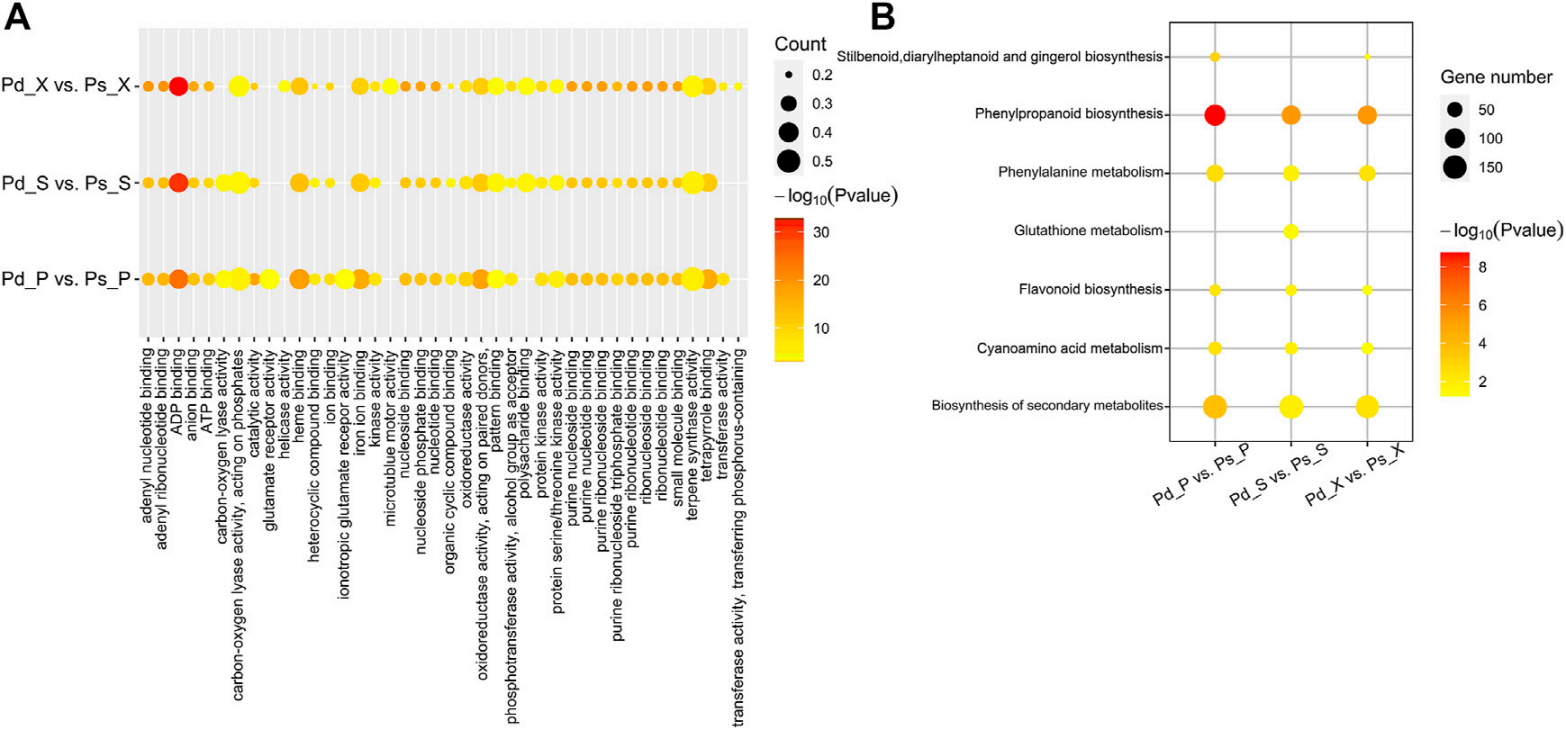
Co-expression mRNA of DElncRANs, GO, and KEGG enrichment. **(A)** GO enrichment analysis of differentially expressed lncRNA-target genes. **(B)** KEGG enrichment analysis of differentially expressed lncRNA-target genes. Node color represents −log10 corrected *p*-value.

The co-expression genes of Pd_ X vs Ps_ X, Pd_ P vs Ps_ P, and Pd_ S vs Ps_ S were mainly related to “ADP binding”, “heme binding”, and other biological functions (Figure 4A). And they were significantly enriched in the “phenylpropanoid biosynthesis” and “cyanoamino acid metabolism” pathway (Figure 4B). These possible target genes provide new insights into the role of lncRNAs in poplar development.

### Regulation of lncRNAs and Transcription Factors in Phenylpropanoid Biosynthesis

**FIGURE 5:**
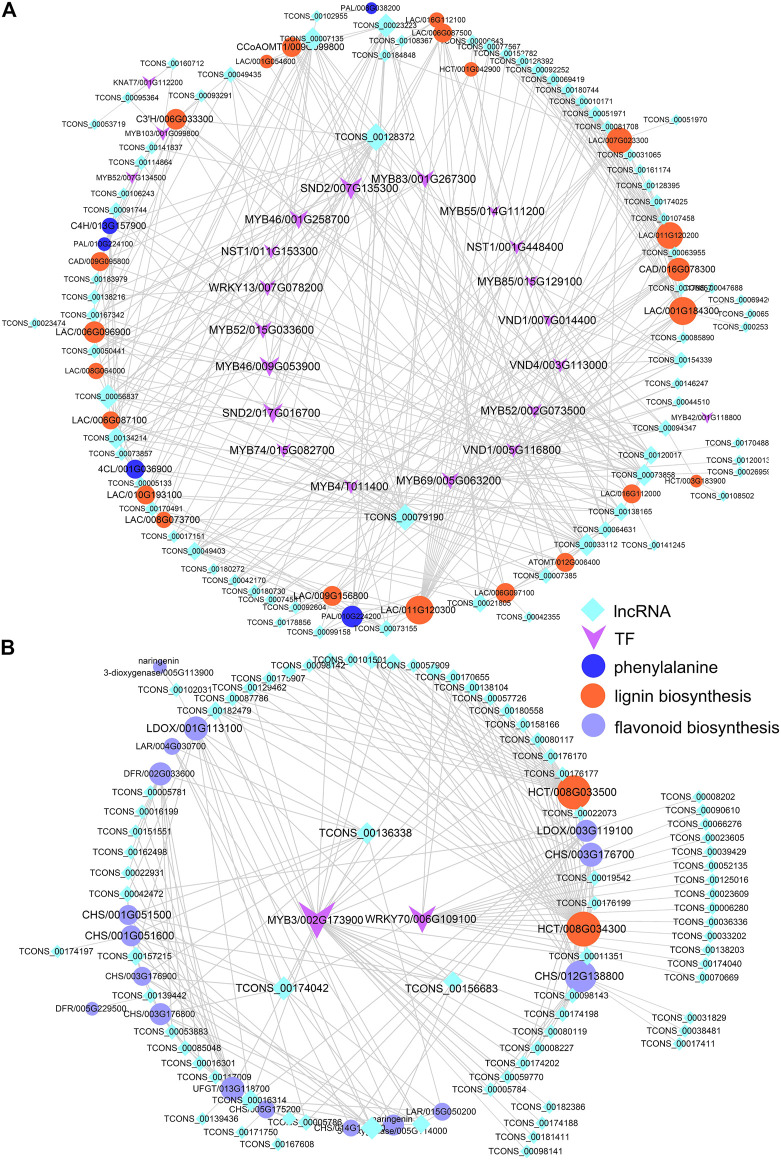
Interaction network diagrams. Interaction networks of differentially expressed lncRNAs, DEmRNAs, and transcription factors (TFs) of phenylalanine biosynthesis, including lignin **(A)** and flavonoid **(B)** biosynthesis.

Based on the predicted GO conditions of growth differential lncRNAs and the pathways associated with target genes, we speculated that lncRNAs might play an important role in phenylalanine biosynthesis in poplar. Phenylpropanoids are a group of plant secondary metabolites derived from phenylalanine, which has a variety of structural and signal molecular functions (Costa et al., 2003). It is the starting compound for biosynthesis of lignin, flavonoids, anthocyanins, etc., and a core mediator of crosstalk between development- and defense-related pathways (Alessandra et al., 2010). Further analysis co-expression network of these lncRNAs, and structure genes of lignins, and flavonoid biosynthesis found that TCONS_00128372, lincRNA, was located in Chr12 and interacted with MYB46, secondary wall–associated NAC domain2 (*SND2*), cinnamate-4-hydroxylase (*C4H*), caffeoyl-CoA 3-O-methyltransferase (*CcoAMT*), and laccase (*LAC*). And sense_overlapping lncRNA TCONS_00079190 co-expressed with *MYB83*, *MYB46*, NAC secondary wall thickening promoting factor1 (*NST1*), and *LAC* (Figure 5A). Similarly, the results of the study by Quan et al. (2019) and Zhou et al. (2017) showed that lncRNAs and miRNAs regulated lignin biosynthesis by regulating TFs such as *bHLHs* and *bZIPs* or directly coexpressing with structural genes in the process of lignin biosynthesis. And TCONS_00174042, TCONS_00101258, and TCONS_00136338 regulated the structural genes of flavonoid biosynthesis. TCONS_00174042 and TCONS_00101258 co-expressed with *MYB3*, chalcone and stilbene synthase (*CHS*), leucoanthocyanidin reductase (*LAR*), leucoanthocyanidin dioxygenase (*LDOX*), and naringenin 3- dioxygenase (*F3H*). In addition, TCONS_00136338 can coexpress with *LDOX*, *CHS*, *UFGT*, *LAR*, and dihydroflavonol 4-reductase (*DFR*) (Figure 5B). Lignin-related lncRNAs and structural genes were highly expressed in xylem of Pd and Ps, while flavonoid-related genes were highly expressed in the SAM (Figure S5).

### lncRNAs Involved in the Plant Hormone Biosynthesis Signal Transduction Pathway

**FIGURE 6:**
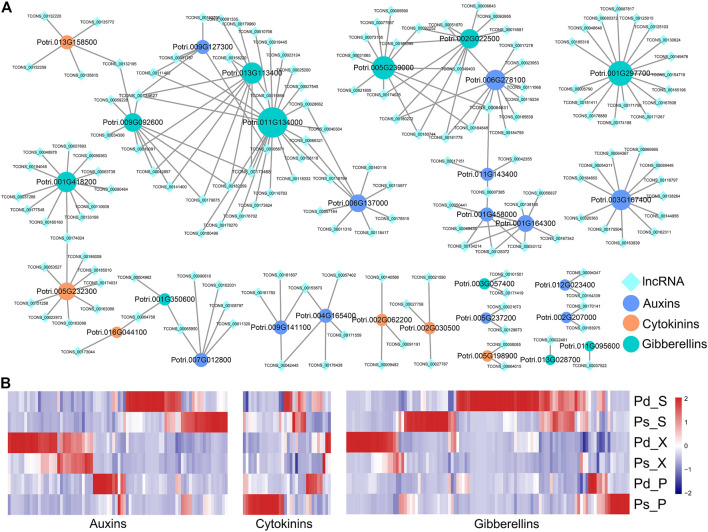
lncRNAs related to plant hormone biosynthesis and signal transduction. **(A)** Co-expression network of DElncRNAs and protein coding genes (PCgenes) involved in auxin, cytokinin, and gibberellin biosynthesis and signal transduction. **(B)** Expression of selected lncRNAs and their predicted co-expression target PCgenes involved in plant hormone biosynthesis signal transduction.

Hormones such as auxin, cytokinin, and gibberellin play important roles in primary and secondary growth (Nieminen et al., 2008; Xu et al., 2019). In order to further analyze the relationship between lncRNAs and plant hormones, we constructed a co-expression network including the structural genes of auxin, cytokinin, and gibberellin biosynthesis and their possible regulatory lncRNAs (Figure 6A). For example, TCONS_00134627 could be coexpressed with *GA2OX8* (Potri.011G134000), *GASA10* (Potri.009G092600), and *SAUR94* (Potri.009G127300) (Figure 6A). The expression levels of DEGs and DElncRNAs related to auxin, cytokinin, and gibberellin biosynthesis were shown in the heatmap (Figure 6B). The differential genes related to auxins and gibberellins were mainly concentrated in the high expression of Pd_S and Pd_X, while only a few genes are highly expressed in the phloem. And the genes related to cytokinins are highly expressed in Ps_P (Figure 6B).

### MicroRNAs Involved in the Hormone and Phenylpropanoid Pathway

**FIGURE 7:**
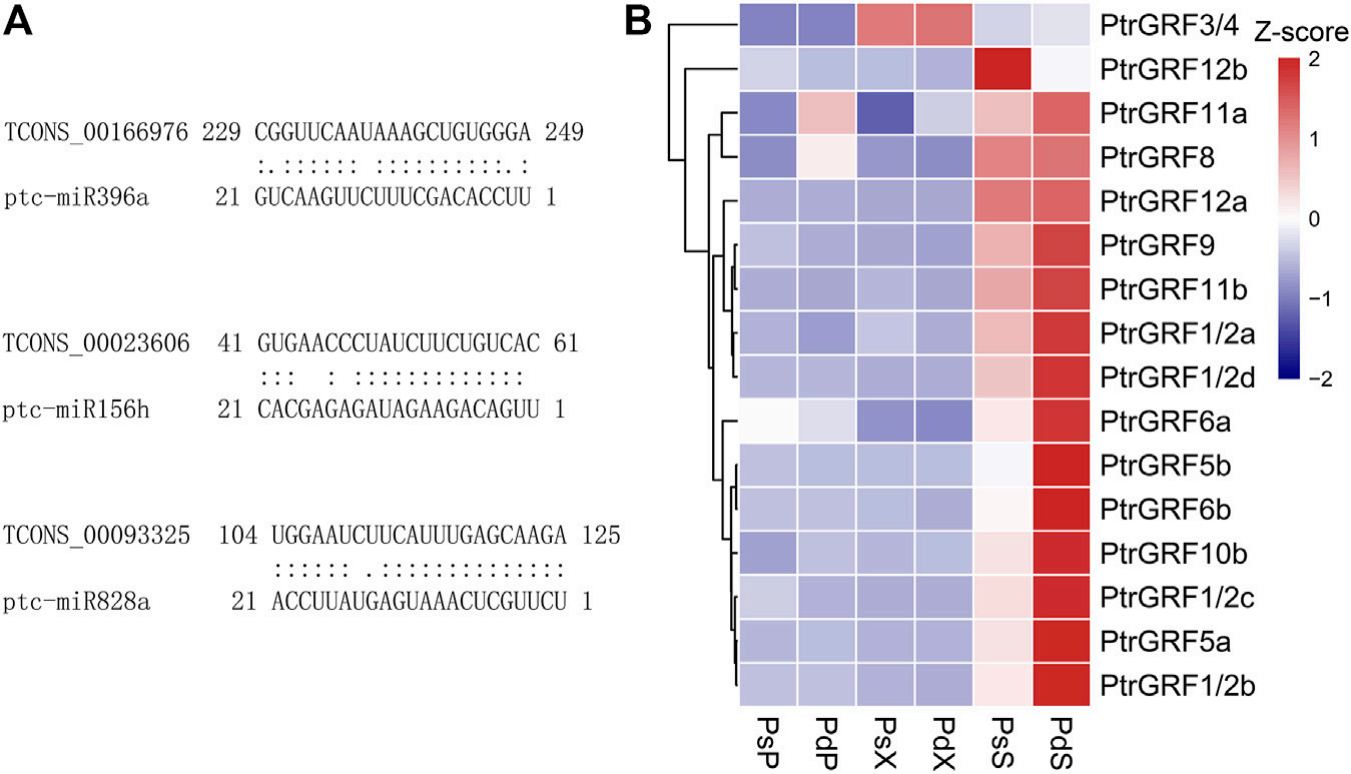
Expression analysis of related lncRNAs as potential targets or target mimics of miRNAs. **(A)** Predicted binding alignment of lncRNAs with ptc-miR396a, ptc-miR156h, and ptc-miR828a. **(B)** Expression patterns of differentially expressed *GRFs and* the target genes of miR396 in the two species.

To further analyze whether lncRNAs were used as ceRNAs to absorb miRNAs, inhibited the effect of miRNAs, and promoted mRNA expression, we conducted sequential analysis. MicroRNAs, a major class of small RNAs with 20–24 nucleotides, create various aspects of plant development and stress responses through posttranscriptionally regulated gene expression (Yu et al., 2019). A total of 658 miRNA–lncRNA pairs consisting of 188 miRNAs and 200 lncRNAs were identified, including 19 plant hormone–related pairs and 28 phenylalanine-related pairs (Supplementary Table 6). TCONS_00066905, hormone-related lncRNA, was predicted to be a target mimic of miR396a and miR396b. In addition, phenylalanine-related TCONS_00023606 and TCONS_00093325 were the target mimics of flavonoid-related regulatory genes miR156h and miR828a (Figure 7A). miR396-*GRF* was an important regulatory module of plant growth and development. We found that 12 *GRFs* were differentially expressed and highly expressed in the SAM of Pd, which may be an important reason for the rapid growth of Pd (Figure 7B).

### Validation of lncRNA and Gene Expression by qRT-PCR

In order to verify the accuracy of transcriptome data, differential genes related to xylem development and hormone signal transduction were selected for qRT-PCR verification. The results showed that the expression trends of three lncRNAs and three mRNAs in six tissues were consistent with those of qRT-PCR, which proved the reliability of transcriptome data (Figure S6).

## Discussion

Poplar is an important bioenergy tree in the world. Its growth rate and wood quality determine its economic value. With the development of RNA-seq technology, genome-wide mapping has been proved to be a powerful tool for studying primary and secondary growth of poplar. Pd and Ps have great differences in plant height, ground diameter, and other growth characters, as well as wood qualities, such as basic density and lignin content (Figure 1). In this study, lncRNAs of woody plants was comprehensively analyzed to study their growth and development and the regulation of wood quality. We identified 6,355 lncRNAs, including 3,572 DElncRNAs and 27, 582 DEGs. lncRNAs in Pd and Ps show similar characteristics with other species (Ci et al., 2019). They are characterized by high tissue specificity and short length. The length of lncRNAs was about 991 bp, and the *cis-* and *trans-* effects were recognized. In this study, we identified a large number of trans-regulatory networks, mainly acting on the phenylalanine pathway. These differentially expressed genes may be an important reason for the differences in growth rate and wood quality.

The SCW provides rigidity and strength for plants to support their body weight and ensure water and nutrient transport (Oda and Fukuda, 2012; Wang and Dixon, 2012). It is mainly composed of lignin, cellulose, and hemicellulose, and their biosynthesis was highly related to the transformation and production of biofuels and biological products (Mohnen, 2008; Hussey et al., 2019). It is regulated by microRNA, *MYB*, *NAC*, and *WRKY* in the SCW complex regulatory network (Zhang et al., 2018b). As an important support to the SCW, lignin determines the conversion efficiency of poplar as biomass energy. In previous studies, lncRNAs participate in the lignin biosynthesis of poplar with TFs and miRNAs (Quan et al., 2019; Zhang et al., 2020c). Also, in our research, we found that 75 lncRNAs, including TCONS_00079190, TCONS_00128372, and TCONS_00007135, can be directly coexpressed with *MYB*s, *VND*s, and lignin structural genes (Figure 5A). Flavonoids and lignin are the metabolic pathways of phenylalanine metabolism, and there are some common structural genes. At the same time, flavonoids are important compounds for plants to respond to biological and abiotic stresses. lncRNAs are involved in regulating the anthocyanin biosynthetic pathways in strawberry, buckthorn, and apple (Zhang et al., 2018a; Lin et al., 2018; Yang et al., 2019). We identified a large number of lncRNAs coexpressed with structural genes of the flavonoid pathway, such as *CHS* and *DFR* (Figure 5B). The insertion of five *cis*genes encoding gibberellin metabolism or signal proteins affects plant growth (Han et al., 2011). The auxin-mediated Aux/IAA-ARF-HB signal cascade regulates the development of the secondary xylem of poplar (Xu et al., 2019). And it was found that a large number of auxin- and gibberellin-related lncRNA–mRNA coexpression networks were identified (Figure 6), which was similar to endogenous hormone regulation in the secondary xylem and during tension wood formation in *Catalpa bungei* (Xiao et al., 2020). Therefore, lncRNAs were widely involved in lignin and flavonoid metabolism and plant growth of Pd and Ps, and they affect their differences.

Posttranscriptional regulation is an important process affecting gene expression and plant development (Li and Zhang, 2016; Yu et al., 2019). miR156 and miR828 are involved in the biosynthesis of flavonoids and anthocyanins by regulating *MYB*s in many species (Tirumalai et al., 2019; B. Zhang et al., 2020a; Wang et al., 2020b). We identified TCONS_00023606 and TCONS_00093325 as the target genes of miRNA156 and miR828, respectively, so they may indirectly participate in the transcriptional regulation of flavonoids through this pathway (Figure 7; Supplementary Table 3). miR396, *GRF*s, and GRF-INTERACTING FACTORS (*GIF*s) have been proven to control the growth of multiple tissues and organs of multiple species (Debernardi et al., 2014; Liebsch and Palatnik, 2020). *GRF*s are important regulators of the SAM, which are the starting sites of leaf and stem development (Wang Y. et al., 2020). The high expression of *GRFs* in Pd_S may be an important reason for the rapid growth of Pd. lncRNAs–miRNAs–TFs–mRNAs play an important role in regulating the growth of poplars. Therefore, the differences in the growth rate and wood quality of the two poplars may be caused by the joint regulation of these factors, which requires our follow-up for further functional verification.

## Data Availability

The datasets presented in this study can be found in online repositories. The names of the repository/repositories and accession number(s) can be found in the article/Supplementary Material.

## Supplementary Material

Supplementary Figure 1 Differentially expressed mRNAs (DEGs) in pair-wise comparison of 18 samples. Number of different genes **(A)** and heat maps **(B)** of different combinations were compared. **(C)** Gene Ontology (GO) enrichment of DEGs in different comparisons. It is detailed enrichment of the biological process (BP). Node color represents −log10 corrected *p*-value.

Supplementary Figure 2 GO enrichment of lncRNA colocation target genes in nine comparative combinations.

Supplementary Figure 3 Expression profile of lncRNAs and mRNAs related to photosynthesis in different tissues.

Supplementary Figure 4 GO **(A)** and KEGG **(B)** analyses of co-location and co-expression mRNAs. BP, biological process; MF, molecular function; CC, cellular component.

Supplementary Figure 5 Expression patterns of lncRNAs **(A)** and mRNAs **(B)** related to lignin and flavonoid biosynthesis.

Supplementary Figure 6 lncRNA and mRNA results from RNA-seq verified by qRT-PCR. Mean ± SD from three biological replicates.

Supplementary Table 1 Primer pairs for qRT- PCR.

Supplementary Table 2 RNA-seq filter data.

Supplementary Table 3 Basic information of lncRNAs was identified in *P*. *deltoides* “Danhong” and *P*. *simonii* “Tongliao1.”

Supplementary Table 4 Co-location target mRNAs of lncRNAs.

Supplementary Table 5 Co-expression mRNAs of lncRNAs.

Supplementary Table 6 Phenylpropanoid biosynthesis–related lncRNAs targeted by miRNAs.
