# Supplementary figures and images for: Genome-Wide Identification of Long Non-Coding RNAs and Their Potential Functions in Poplar Growth and Phenylalanine Biosynthesis
Source: Front Genet. 2021 Nov 15;12:762678. doi: 10.3389/fgene.2021.762678 (PMC8634849; doi:10.3389/fgene.2021.762678)

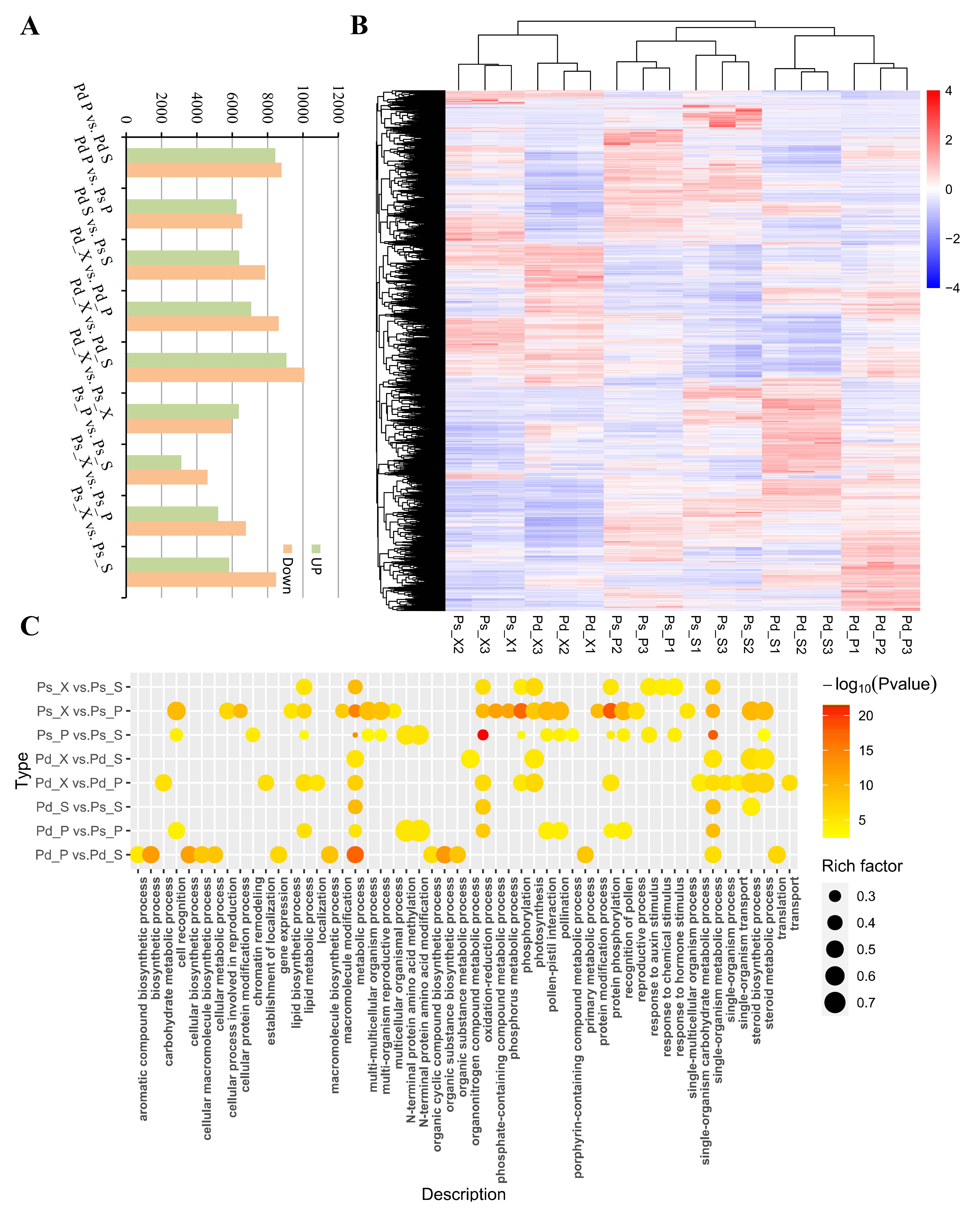

Supplement: Supplementary file 2 [file Image1.JPEG]

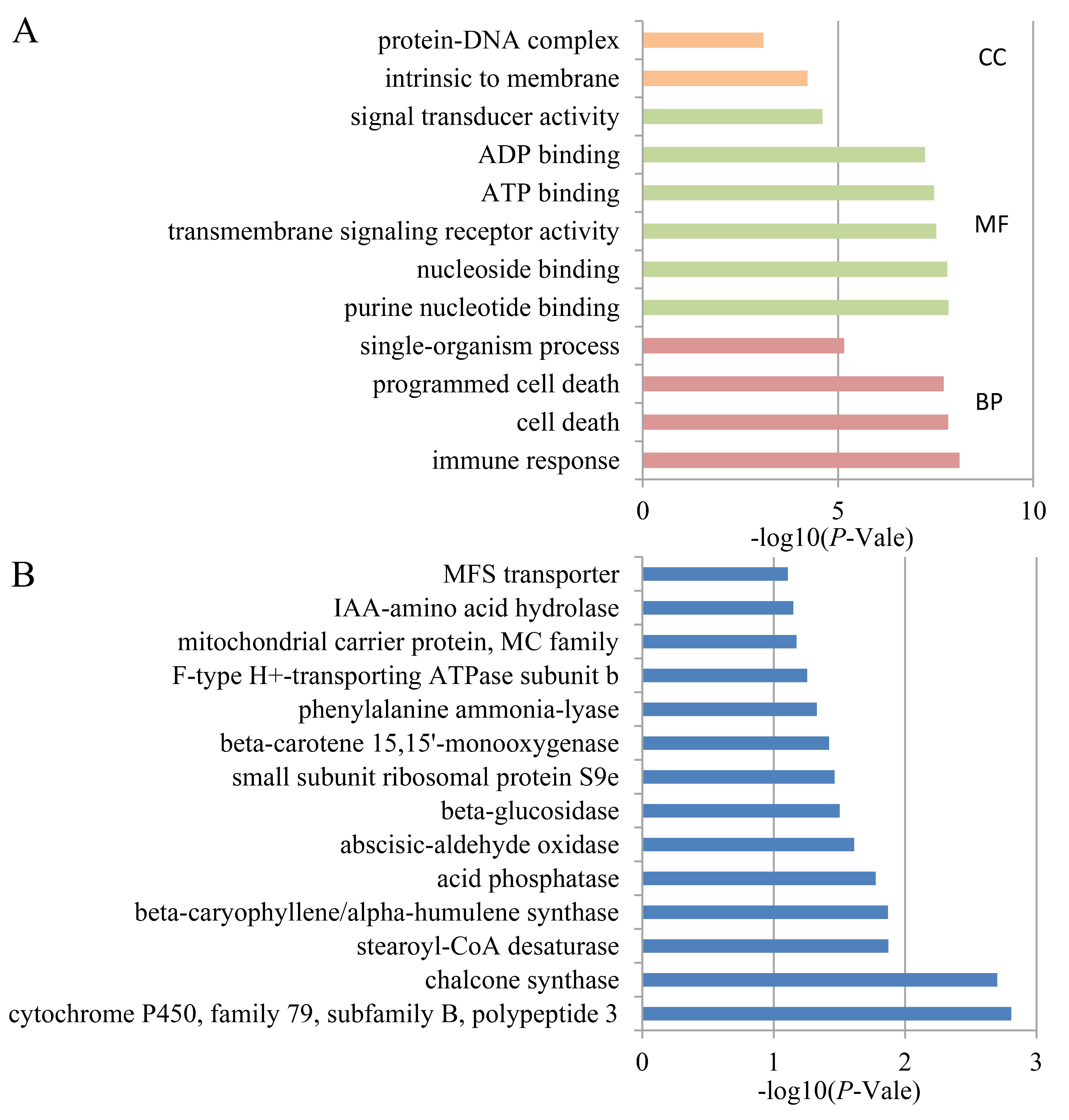

Supplement: Supplementary file 3 [file Image4.JPEG]

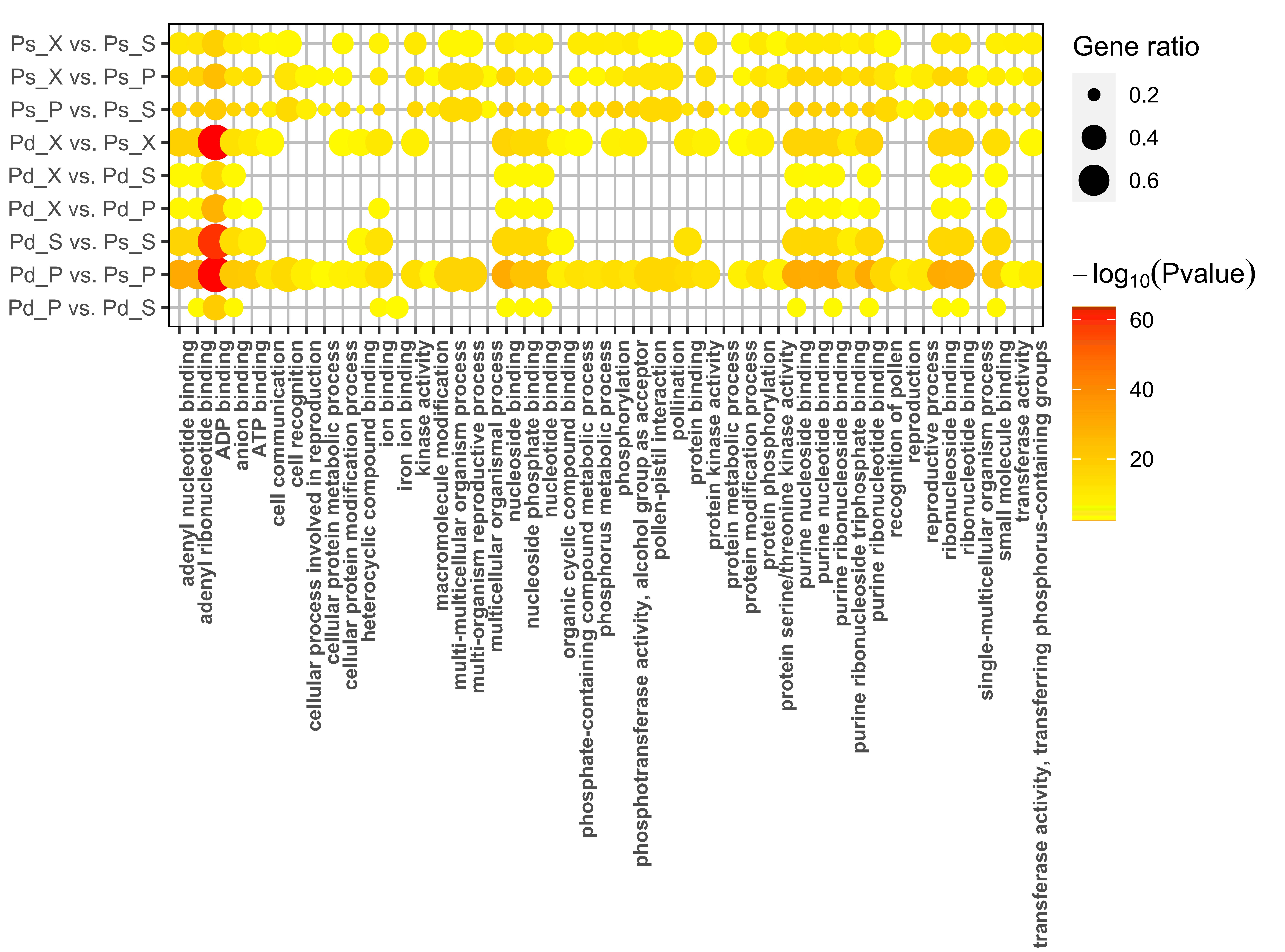

Supplement: Supplementary file 4 [file Image2.JPEG]

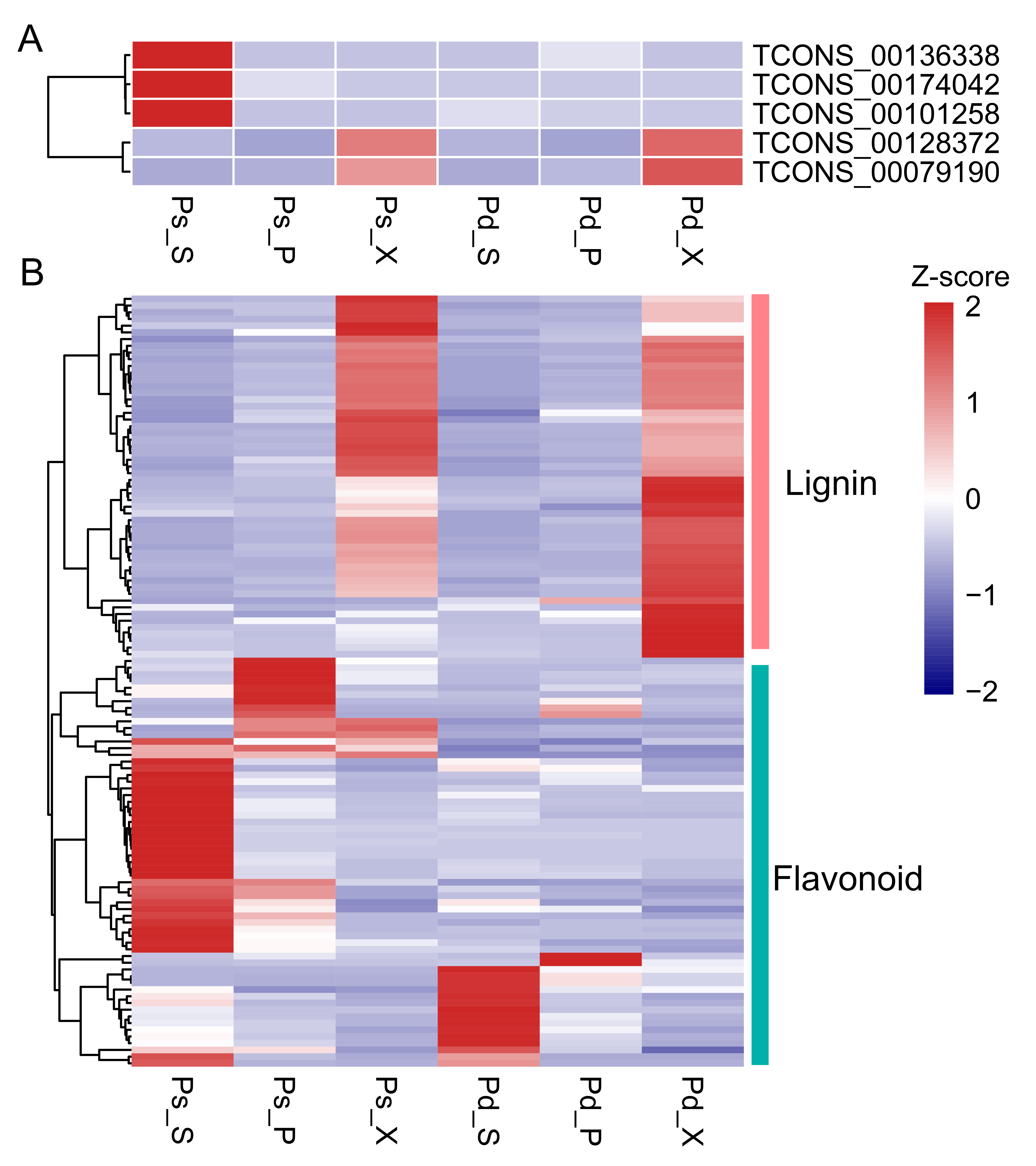

Supplement: Supplementary file 5 [file Image5.JPEG]
